# Exogenous Melatonin Improves Drought Tolerance by Regulating the Antioxidant Defense System and Photosynthetic Efficiency in Fodder Soybean Seedings

**DOI:** 10.3390/plants14030460

**Published:** 2025-02-05

**Authors:** Qianhan Zhao, Xueling Zheng, Chen Wang, Qinyi Wang, Qiyun Wei, Xiashun Liu, Yujiao Liu, Along Chen, Jia Jiang, Xueying Zhao, Tiantian He, Jiayi Qi, Yuchen Han, Haonan Qin, Fuchun Xie, Yajun Chen

**Affiliations:** 1 College of Animal Science and Technology, Northeast Agricultural University, Harbin 150030, China; s230502043@neau.edu.cn (Q.Z.);; 2 College of Horticulture, Northeast Agricultural University, Harbin 150030, China; zxue2002@outlook.com (X.Z.);; 3 Fujian Zhongke Biological Co., Ltd., Xiamen 361001, China

**Keywords:** fodder soybean, drought stress, melatonin, photosynthesis, antioxidant system

## Abstract

Fodder soybean (*Glycine max* L.) with high protein and yield is a popular forage grass in northeast China. Seasonal drought inhibits its growth and development during seedling stage. The objective of this study was to observe morpho-physiological changes in fodder soybean seedlings under melatonin (MT) treatments and identify appropriate concentration to alleviate the drought damage. Two varieties commonly used in northeast China were treated with 0, 50, 100, and 150 μM melatonin at soil water content of 30%. The results indicated that applying melatonin enhanced height, biomass and altered root morphology of fodder soybean seedlings under water-deficient conditions. The treatments with melatonin at different concentrations significantly reduced the contents of H_2_O_2_, O_2_^−^ and MDA, while boosting the capacity of the antioxidant defense system and the content of osmotic adjustment substances. Meanwhile, increases in light energy capture and transmission efficiency were observed. Furthermore, treatment with melatonin regulated the expression levels of genes associated with photosynthesis and the antioxidant defense system. Notably, 100 μM melatonin treatment produced the most favorable effect in all treatments under drought conditions. These research results provide new information for enhancing the drought tolerance of fodder soybean using chemical measures.

## 1. Introduction

Drought ranks among the most detrimental abiotic stresses, impacting plant growth, development, and yield. Severe drought may accelerate senescence and even lead to death [1,2]. Under drought conditions, plants undergo a range of phenotypic and physiological changes, including inhibiting growth, wilting the leaves, adjusting root architecture [3], disrupting photosynthesis [4], accumulating reactive oxygen species (ROS), and altering cellular membrane permeability [5,6]. To mitigate the negative influences of drought on plants, extensive research has been conducted to enhance drought tolerance. Previous studies have shown that exogenous hormone application can effectively improve plant adaptability to drought stress [7], but the responses of different plants to exogenous hormone types and concentrations are inconsistent. This indicates that selecting the appropriate plant hormone and concentration is vital to enhancing plant adaptive capacity in water-deficient environments.

Melatonin (MT) is an indoleamine compound widely present in plants [8], associated with various physiological and metabolic processes, such as seed germination [9], flowering and fruiting [10], as well as senescence [11]. Moreover, melatonin plays a crucial role in regulating plant adaptation to stress environments [12,13]. Especially under drought stress, melatonin can promote shoot growth and root development [14,15], enhance the antioxidant capacity of plants, remove ROS due to drought and alleviate oxidative damage, thereby improving drought tolerance [16,17,18]. Previous studies have indicated that exogenous melatonin alleviates drought-induced damage to cell membranes by regulating antioxidant defense systems and osmotic regulatory substances in crops such as rice (*Oryza sativa* L.) [19], potato (*Solanum tuberosum* L.) [20] and maize (*Zea mays* L.) [21]. Additionally, melatonin protects photosynthetic organs from drought-induced damage, such as slowing chlorophyll degradation during drought stress, improving light absorption and electron transport capacities [18,22]. In wheat (*Triticum aestivum* L.), application of melatonin significantly increased net photosynthetic rate (Pn), photosystem II efficiency (Fv/Fm) and the content of GSH and AsA under drought stress [23]. These findings suggest a close relationship between exogenous melatonin and plant physiological response to drought. However, the optimal melatonin concentration for enhancing drought tolerance of fodder soybean (*Glycine max* L.) remains unclear [24].

Fodder soybean is an annual herbaceous plant in the Fabaceae family, characterized by its soft stems, abundant branches, and high protein content. It is easy to cultivate and has a palatable taste, making it an excellent choice for high-quality forage crops [25,26]. However, fodder soybean seedlings require substantial water for growth, and severe spring drought can notably hinder the growth, adversely affecting its yield and nutritional value [27]. Therefore, it is extremely important to search effective measures of maintaining the normal growth of fodder soybeans under drought conditions to ensure the quality and yield of fodder soybeans. Fodder soybean seedlings experiencing drought stress were applied with different concentrations of melatonin, and changes in morphological and physiological parameters were investigated with a view to determine the optimal concentration of melatonin suitable for protecting soybean seedlings during drought stress condition. These findings provide a theoretical reference for improving fodder soybean quality and yield under drought conditions in future production.

## 2. Results

### 2.1. Effects of Different Melatonin Concentrations on Growth of Fodder Soybean Under Drought Stress

Drought led to visible signs of water loss such as curling and wilting of leaves (Table S1) and plants of shorter statue. However, treatment with melatonin of different concentrations in a water-deficient environment alleviated leaves’ wilting (Figure S1) and had a positive effect on plant height of fodder soybeans (Table 1). Under normal growth conditions, applying 50 μM, 100 μM and 150 μM of melatonin to ‘Mudanjiang’ fodder soybean significantly increased plant height by 4.78%, 8.99% and 5.47%, respectively, compared to applying water, while no significant increase was observed for the ‘Gongnong 535’ fodder soybean (Table 1). However, the plant height of ‘Gongnong 535’ fodder soybean was markedly increased by 7.83% with the 100 μM melatonin treatment compared to treatment with water under drought stress (Table 1). Simultaneously, significant increases in plant height were observed with 50 μM and 100 μM melatonin treatments for ‘Mudanjiang’ fodder soybean, with increases of 6.18% and 9.12%, respectively (Table 1).

Melatonin treatments regulated the biomass of fodder soybeans. Foliar application of 100 μM and 150 μM melatonin in the normal water group observably increased the aboveground biomass of ‘Gongnong 535’ fodder soybean by 4.17% and 3.82% compared to treatment with water, while the increase in belowground biomass was not statistically significant (Table 1). For ‘Mudanjiang’ fodder soybean, treatment with 100 μM melatonin resulted in increasing both aboveground and belowground biomass by 14.46% and 15, respectively (Table 1).

Under drought conditions, applying 100 µM melatonin increased both aboveground and belowground biomass of ‘Gongnong 535’ fodder soybean by 8.75% and 11.11%, respectively, compared to treatment with water (Table 1). The aboveground biomass of ‘Mudanjiang’ fodder soybeans increased with the melatonin concentration, and the maximum increase was 15.32% under the concentration of 100 μM (Table 1). The 50 μM, 100 μM and 150 μM melatonin treatments also remarkably enhanced belowground biomass in ‘Mudanjiang’ fodder soybean by 12.12%, 18.18% and 12.12%, respectively (Table 1).

### 2.2. Effects of Different Melatonin Concentrations on Root System of Fodder Soybean Under Drought Stress

**Figure 1:**
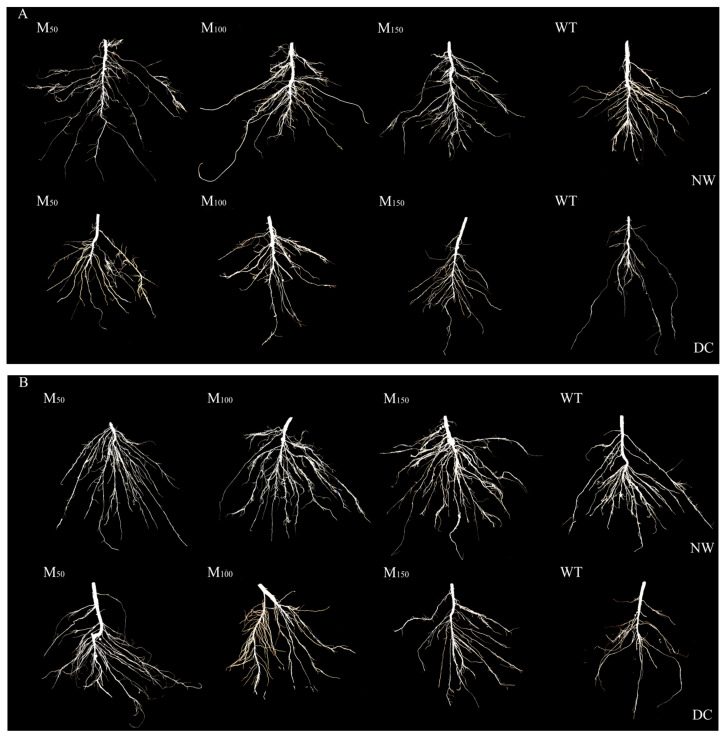
Effects of different melatonin concentrations on root morphology of fodder soybean under drought stress. (**A**) ‘Gongnong 535’ fodder soybean; (**B**) ‘Mudanjiang’ fodder soybean. Abbreviations and their full forms are provided as follows: NW: normal water supply; DC: drought condition; WT: treated with water; M_50_: treated with 50 μM MT; M_100_: treated with 100 μM MT; M_150_: treated with 150 μM MT.

Drought stress significantly affected root morphology and root growth of both fodder soybean cultivars. And application of different melatonin concentrations had obvious effects on the root systems of both cultivars (Figure 1 and Table 2). Under normal conditions, the root surface area of ‘Gongnong 535’ fodder soybean increased by 6.92% after receiving 100 µM melatonin, while that of ‘Mudanjiang’ fodder soybean increased by 6.91% and 5.96% after receiving 50 µM and 100 µM melatonin, respectively (Table 2). Under drought conditions, the root length of ‘Gongnong 535’ fodder soybean treated with 50 μM, 100 μM and 150 μM melatonin was, respectively, 1.27, 1.34 and 1.31 times that of fodder soybean treated with water (Table 2), while for ‘Mudanjiang’ fodder soybean, the greatest increase in root length was observed with the 100 μM treatment, showing a 33.02% growth compared to when applied with water in all treatments. These results indicate that the 100 μM melatonin concentration had the most pronounced positive impact on root growth of fodder soybean under drought stress.

### 2.3. Effects of Different Melatonin Concentrations on Chlorophyll Content and Photosynthetic Capacity of Fodder Soybean Under Drought Stress

**Figure 2:**
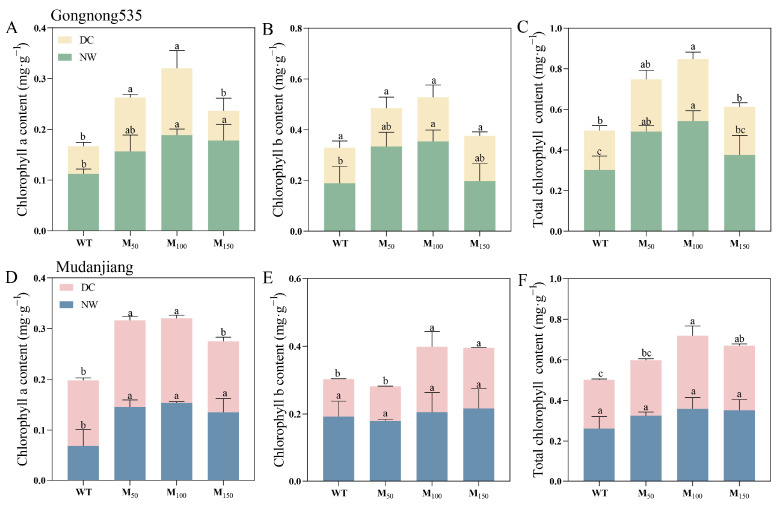
Effects of different melatonin concentrations on photosynthetic pigments of fodder soybean under drought stress. (**A**–**C**) ‘Gongnong 535’ fodder soybean; (**D**–**F**) ‘Mudanjiang’ fodder soybean. Distinct lowercase letters indicate significant differences (*p* < 0.05) among treatments within the same cultivar and watering condition. Vertical bars denote the standard deviation of the mean (*n* = 3). Abbreviations and their full forms are provided as follows: NW: normal water supply; DC: drought condition; WT: treated with water; M_50_: treated with 50 μM MT; M_100_: treated with 100 μM MT; M_150_: treated with 150 μM MT.

The contents of chlorophyll a (Chl a), chlorophyll b (Chl b) and total chlorophyll in fodder soybean were reduced by drought stress. Application of various melatonin concentrations mitigated the negative impact of the water shortage condition on photosynthetic pigments in fodder soybean. Different melatonin concentrations raised Chl a, Chl b and total chlorophyll contents in ‘Gongnong 535’ fodder soybean under both normal watering and drought conditions (Figure 2). The Chl a content of ‘Gongnong 535’ fodder soybean under drought stress reached pronounced levels with 50 μM and 100 μM melatonin applications, 1.96 and 2.42 times that of fodder soybean treated with water, respectively (Figure 2A). And for ‘Mudanjiang’ fodder soybean under normal conditions, 50 μM, 100 μM and 150 μM MT increased Chl a content, but similar increases were not found for Chl b and total Chl (Figure 2A–C). Under drought conditions, treatment with 100 μM MT increased Chl a, Chl b and total Chl in ‘Mudanjiang’ fodder soybean by 29.12%, 74.17% and 49.93%, respectively, compared with the water treatment (Figure 2A–C). Moreover, according to the results, under drought stress, the total chlorophyll content of ‘Mudanjiang’ fodder soybean with 50 μM, 100 μM and 150 μM melatonin treatments was 5.95%, 17.76% and 34.84% higher than that of ‘Gongnong 535’ fodder soybean, respectively (Figure 2C,F).

**Figure 3:**
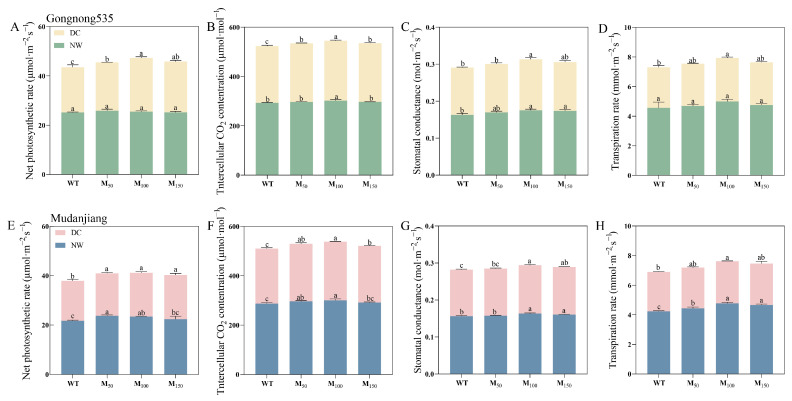
Effects of different melatonin concentrations on photosynthetic indexes of fodder soybean under drought stress. (**A**–**D**) ‘Gongnong 535’ fodder soybean; (**E**–**H**) ‘Mudanjiang’ fodder soybean. Distinct lowercase letters indicate significant differences (*p* < 0.05) among treatments within the same cultivar and watering conditions. Vertical bars denote the standard deviation of the mean (*n* = 3). Abbreviations and their full forms are provided as follows: NW: normal water supply; DC: drought condition; WT: treated with water; M_50_: treated with 50 μM MT; M_100_: treated with 100 μM MT; M_150_: treated with 150 μM MT.

Various concentrations of melatonin increased the values of Pn, Ci, Gs and Tr in ‘Gongnong 535’ fodder soybean under normal watering conditions compared with treatment with water (Figure 3A–D). Notably, Ci showed an increase of 2.71% with the 100 μM treatment, and Gs rose by 8.02% and 6.79% under 100 μM and 150 μM treatments, respectively (Figure 3B,C). While the 50 μM and 100 μM melatonin treatments in ‘Mudanjiang’ fodder soybean enhanced Pn by 9.36% and 7.84%, and Ci by 3.23% and 4.50% (Figure 3E,F). Additionally, Gs increased by 4.49% and 2.56%, and Tr significantly improved by 12.85% and 10.25% with 100 μM and 150 μM treatments (Figure 3G,H). All three melatonin concentrations enhanced photosynthetic parameters to varying extents in ‘Gongnong 535’ soybean under drought stress, with the most significant increases in Pn, Ci, Gs, and Tr observed in the 100 μM treatment (Figure 3A–D). And the most pronounced changes in Ci, Gs and Tr in ‘Mudanjiang’ fodder soybean were seen under the 100 μM treatment, while Pn augmented with 50 μM, 100 μM and 150 μM melatonin treatments showed increases by 6.67%, 9.83% and 10.44%, respectively (Figure 3E–H). The comparison between two varieties under drought conditions showed that under the treatment of three concentrations of melatonin, Pn of ‘Gongnong 535’ was 13.62%, 22.09% and 15.38% higher than that of ‘Mudanjiang’, while the difference in other photosynthetic indexes was not obvious (Figure 3).

### 2.4. Effects of Different Melatonin Concentrations on Chlorophyll Fluorescence of Fodder Soybean Under Drought Stress

**Figure 4:**
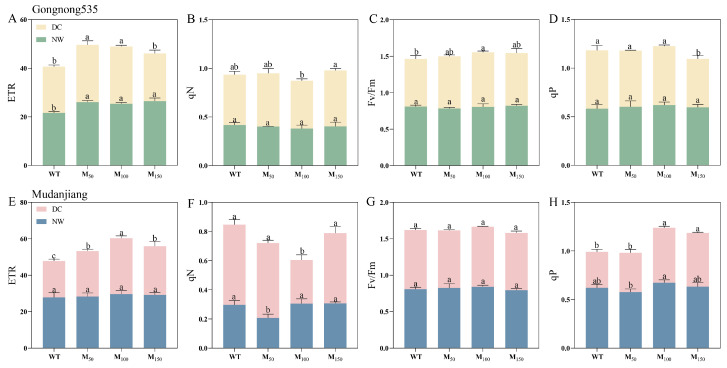
Effects of different melatonin concentrations on chlorophyll fluorescence of fodder soybean under drought stress. (**A**–**D**) ‘Gongnong 535’ fodder soybean; (**E**–**H**) ‘Mudanjiang’ fodder soybean. Distinct lowercase letters indicate significant differences (*p* < 0.05) among treatments within the same cultivar and watering conditions. Vertical bars denote the standard deviation of the mean (n = 3). Abbreviations and their full forms are provided as follows: NW: normal water supply; DC: drought condition; WT: treated with water; M_50_: treated with 50 μM MT; M_100_: treated with 100 μM MT; M_150_: treated with 150 μM MT.

Melatonin treatments significantly improved the fluorescence parameters of fodder soybean under different soil water conditions. Under normal water supply conditions, the application of melatonin at 50 μM, 100 μM and 150 μM enhanced ETR of ‘Gongnong 535’ fodder soybean by 20.34%, 17.41% and 22.19%, respectively, compared to treatment with water (Figure 4A), while 50 μM melatonin treatment significantly decreased qN by 30.34% compared to treatment with water in ‘Mudanjiang’ fodder soybean under normal watering conditions (Figure 4F). Under drought stress, the ETR of ‘Gongnong 535’ fodder soybean increased with 50 μM and 100 μM melatonin treatments, both showing an enhancement of 23.82% compared to treatment with water (Figure 4A). It also showed a notable increase in Fv/Fm by 14.82% with the 100 μM treatment, while qP decreased by 16.78% with the 150 μM melatonin treatment. For ‘Mudanjiang’ fodder soybean under drought stress, the 100 μM melatonin treatment for ETR and qN resulted in the most significant change, with increases of 53.67% and 45.48% compared to treatment with water, respectively (Figure 4E–H). The qP parameter showed a higher improvement with 100 μM and 150 μM melatonin than other treatments, increasing 52.79% and 49.10%, respectively (Figure 4H). These findings indicate that the application of varying melatonin concentrations under drought stress significantly influenced the chlorophyll fluorescence parameters in both fodder soybean cultivars (Figure 4).

### 2.5. Effects of Different Melatonin Concentrations on Osmotic Adjustment Substances in Fodder Soybean Under Drought Stress

**Figure 5:**
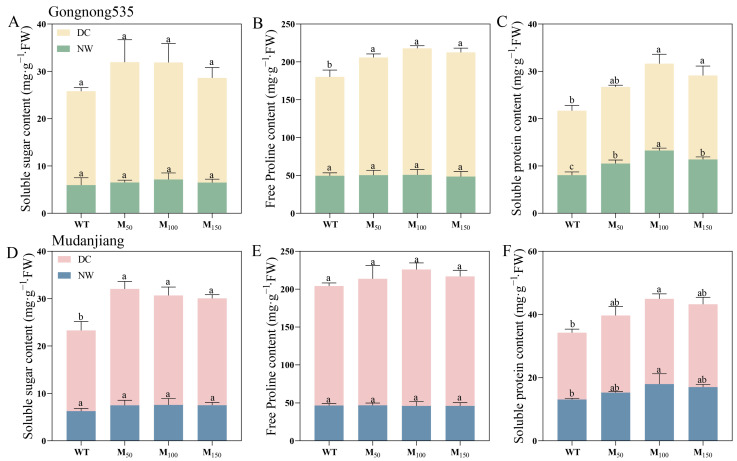
Effects of different melatonin concentrations on osmotic regulatory substances of fodder soybean under drought stress. (**A**–**C**) ‘Gongnong 535’ fodder soybean; (**D**–**F**) ‘Mudanjiang’ fodder soybean. Distinct lowercase letters indicate significant differences (*p* < 0.05) among treatments within the same cultivar and watering condition. Vertical bars denote the standard deviation of the mean (*n* = 3). Abbreviations and their full forms are provided as follows: NW: normal water supply; DC: drought condition; WT: treated with water; M_50_: treated with 50 μM MT; M_100_: treated with 100 μM MT; M_150_: treated with 150 μM MT.

Different concentrations of melatonin had no notable effect on SS or Pro content in both fodder soybean cultivars under normal watering conditions (Figure 5A,B,D,E). SP content with 100 μM MT treatment showed the most noteworthy change, increasing by 64.42% and 37.46% in ‘Gongnong 535’ and ‘Mudanjiang’ fodder soybean under normal water conditions (Figure 5C,F). However, under drought stress, application of MT significantly increased osmotic adjustment substances. Among them, the Pro content in ‘Gongnong 535’ fodder soybean with 50 μM, 100 μM and 150 μM MT treatments was enhanced by 19.11%, 28.00% and 25.65%, respectively, compared to fodder soybean treated with water (Figure 5B). Meanwhile, after treatment with 50 μM, 100 μM and 150 μM MT concentrations, the SS content of ‘Mudanjiang’ fodder soybean increased by 44.33%, 35.70% and 32.56% compared to water treatment (Figure 5D). Moreover, the 100 μM melatonin treatment resulted in the SP content of ‘Gongnong 535’ and ‘Mudanjiang’ fodder soybean under drought condition being 1.35 and 1.30 times that of fodder soybean treated with water (Figure 5C,F). Moreover, the change in SP content in ‘Gongnong 535’ fodder soybean also reached a remarkable level when 150 μM melatonin was applied, which was 1.35 times that of fodder soybean treated with water (Figure 5C). Under drought condition, the Pro content of ‘Mudanjiang’ fodder soybean treated with 50 μM, 100 μM and 150 μM MT was 50.63%, 46.76% and 47.50% higher than that of ‘Gongnong 535’ fodder soybean, respectively (Figure 5B,E).

### 2.6. Effects of Different Melatonin Concentrations on ROS Accumulation in Fodder Soybean Under Drought Stress

**Figure 6:**
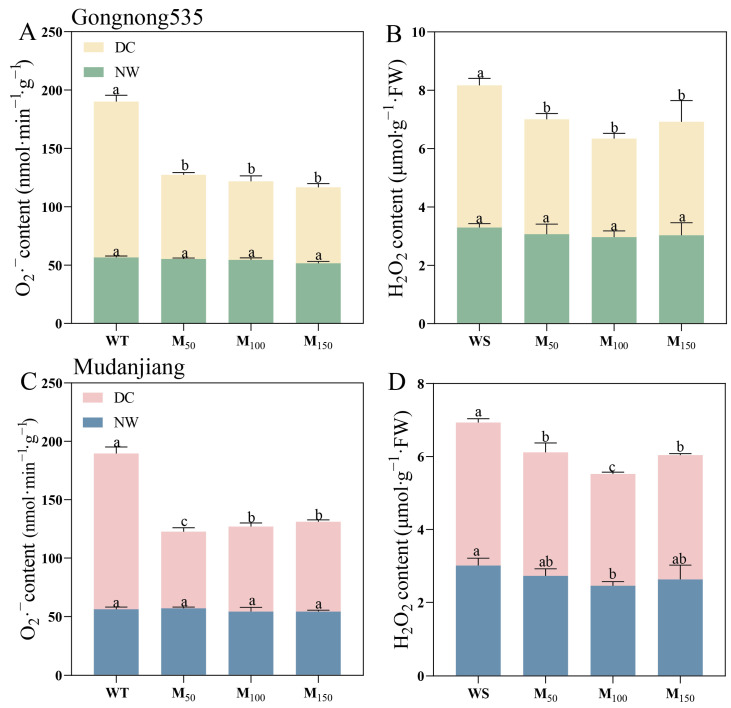
Effects of different melatonin concentrations on ROS accumulation in fodder soybean under drought stress. (**A**,**B**) ‘Gongnong 535’ fodder soybean; (**C**,**D**) ‘Mudanjiang’ fodder soybean. Distinct lowercase letters indicate significant differences (*p* < 0.05) among treatments within the same cultivar and watering condition. Vertical bars denote the standard deviation of the mean (*n* = 3). Abbreviations and their full forms are provided as follows: NW: normal water supply; DC: drought condition; WT: treated with water; M_50_: treated with 50 μM MT; M_100_: treated with 100 μM MT; M_150_: treated with 150 μM MT.

Drought stress led to elevated levels of H_2_O_2_ and O_2_^−^ in plant leaves, whereas the application of various melatonin concentrations markedly decreased ROS accumulation (Figure 6). Melatonin application had an effect on the H_2_O_2_ content in ‘Mudanjiang’ fodder soybean under normal water conditions, but not significantly (Figure 6D). Under drought stress, all concentrations of melatonin remarkably decreased H_2_O_2_ and O_2_^−^ levels in both fodder soybean cultivars. For ‘Gongnong 535’ fodder soybean, 50 μM, 100 μM and 150 μM MT treatments decreased O_2_^−^ levels by 84.97%, 98.49% and 105.23% and reduced H_2_O_2_ levels by 23.73%, 44.23% and 25.12%, respectively, compared to treatment with water (Figure 6A,B). Meanwhile, H_2_O_2_ and O_2_^−^ contents decreased significantly in ‘Mudanjiang’ fodder soybean, with application of 50 μM, 100 μM and 150 μM MT. The most pronounced reduction in H_2_O_2_ content (27.80%) was observed with the 100 μM melatonin treatment, while the 50 μM treatment led to the most notable decrease in O_2_^−^ content, with a reduction of 15.71% (Figure 6C,D). Under drought stress, H_2_O_2_ content of ‘Mudanjiang’ fodder soybean with 50 μM,100 μM and 150 μM MT treatments was lower than ‘Gongnong 535’ fodder soybean by 13.94%, 9.17% and 12.42%, respectively (Figure 6B,D).

### 2.7. Effects of Different Melatonin Concentrations on Cell Membrane Permeability in Fodder Soybean Under Drought Stress

**Figure 7:**
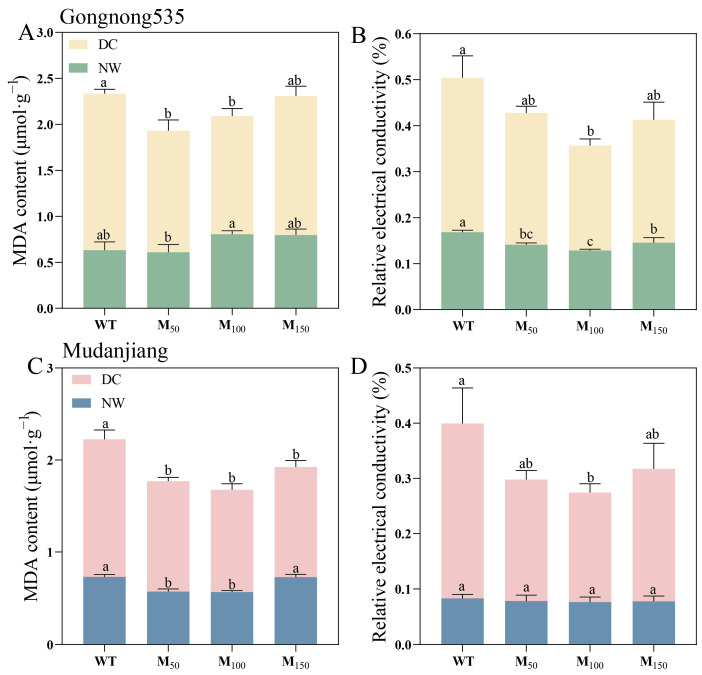
Effects of different melatonin concentrations on membrane permeability of fodder soybean under drought stress. (**A**,**B**) ‘Gongnong 535’ fodder soybean; (**C**,**D**) ‘Mudanjiang’ fodder soybean. Distinct lowercase letters indicate significant differences (*p* < 0.05) among treatments within the same cultivar and watering condition. Vertical bars denote the standard deviation of the mean (*n* = 3). Abbreviations and their full forms are provided as follows: NW: normal water supply; DC: drought condition; WT: treated with water; M_50_: treated with 50 μM MT; M_100_: treated with 100 μM MT; M_150_: treated with 150 μM MT.

Drought stress resulted in a large amount of MDA accumulation and a significant increase in REC, while melatonin application mitigated the impact of drought stress on cell membrane permeability (Figure 7). The MDA content of ‘Gongnong 535’ fodder soybean under normal watering conditions did not show notable changes with any melatonin treatments, while in ‘Mudanjiang’ fodder soybean, MDA content distinctly decreased when treated with 50 μM and 100 μM melatonin. Under drought stress, MDA content in ‘Mudanjiang’ fodder soybean decreased by 24.81%, 34.88% and 24.00% with 50 μM, 100 μM and 150 μM melatonin treatments compared to treatment with water (Figure 7C). For ‘Gongnong 535’ fodder soybean, remarkable reductions in MDA were observed only with 50 μM and 100 μM melatonin, showing declines of 28.53% and 32.51%, respectively (Figure 7A). In terms of REC, different concentrations of melatonin significantly reduced the REC in ‘Gongnong 535’ fodder soybean under normal water conditions compared to treatment with water, whereas no pronounced changes were observed in ‘Mudanjiang’ fodder soybean. Under drought stress, melatonin application decreased REC in both ‘Gongnong 535’ and ‘Mudanjiang’ fodder soybean. Specifically, the 100 μM melatonin treatment resulted in REC reductions of 47.15% and 59.93% in ‘Gongnong 535’ and ‘Mudanjiang’ fodder soybean compared to the water treatment (Figure 7B,D). The MDA content of ‘Gongnong 535’ fodder soybean under drought conditions applied with 50 μM,100 μM and 150 μM MT was higher than that of ‘Mudanjiang’ fodder soybean by 10.16%, 15.84% and 26.20%, respectively (Figure 7A,C).

### 2.8. Effects of Different Melatonin Concentrations on Antioxidant Capacity in Fodder Soybean Under Drought Stress

**Figure 8:**
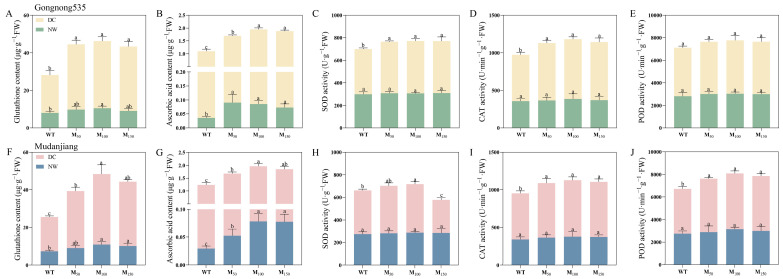
Effects of different melatonin concentrations on ASA, GSH and the activity of antioxidant enzymes contents in fodder soybeans under drought stress. (**A**–**E**) ‘Gongnong 535’ fodder soybean; (**F**–**J**) ‘Mudanjiang’ fodder soybean. Distinct lowercase letters indicate significant differences (*p* < 0.05) among treatments within the same cultivar and watering condition. Vertical bars denote the standard deviation of the mean (*n* = 3). Abbreviations and their full forms are provided as follows: NW: normal water supply; DC: drought condition; WT: treated with water; M_50_: treated with 50 μM MT; M_100_: treated with 100 μM MT; M_150_: treated with 150 μM MT.

The application of various melatonin concentrations enhanced AsA, GSH contents, and the activities of SOD, POD and CAT in fodder soybean under different soil water conditions. Under normal watering condition, GSH content in ‘Gongnong 535’ fodder soybean treated with 100 μM MT was significantly improved by 31.01% compared to treatment with water, while AsA content was significantly increased at all melatonin concentrations, reaching 2.57, 2.42 and 2.07 times that of the water treatment (Figure 8A,B). In ‘Mudanjiang’ fodder soybean, both GSH and AsA contents showed notable enhancement under 100 μM and 150 μM melatonin treatments, with GSH content rising by 48.2% and 38.04%, and AsA content reaching 2.67 and 2.65 times that of treatment with water (Figure 8F,G). Under drought condition, the melatonin application remarkably enhanced GSH and AsA contents in ‘Gongnong 535’ fodder soybean compared to that treated with water, with GSH levels increasing by 72.75%, 77.48% and 70.30%, AsA content rising by 50.74%, 75.03% and 69.45% with 50 μM, 100 μM and 150 μM melatonin treatments, respectively (Figure 8A,B). For ‘Mudanjiang’ fodder soybean, the 100 μM melatonin treatment in all treatments had the most pronounced effects on GSH and AsA contents under drought condition, increasing to 2.06 and 1.55 times those of fodder soybean treated with water (Figure 8F,G).

Different melatonin concentrations did not significantly affect SOD, CAT or POD activities in either fodder soybean cultivar under the normal growing condition. However, under drought stress, application of melatonin at all concentrations in ‘Gongnong 535’ fodder soybean notably increased SOD activity by 13.88%, 15.89% and 14.99% and CAT activity by 23.47%, 28.74% and 25.34%, respectively, but POD activity did not reach prominent levels (Figure 8C–E). For ‘Mudanjiang’ fodder soybean under drought stress, SOD activity enhanced by 11.53% with the 100 μM melatonin treatment but decreased by 24.33% with the 150 μM treatment (Figure 8H). The CAT and POD activities showed a significant enhancement across all melatonin concentrations. Particularly, CAT activity improved by 18.73%, 22.79% and 19.97%, while POD activity was raised by 19.25%, 24.48% and 22.58% with 50 μM, 100 μM and 150 μM melatonin treatments, respectively, compared to water treatment (Figure 8I,J).

### 2.9. Effects of Different Melatonin Concentrations on the Expression Levels of Key Genes Under Drought Stress

**Figure 9:**
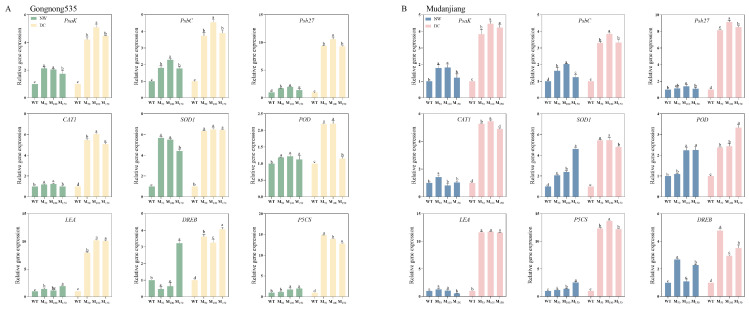
Effects of different melatonin concentrations on relative gene expression in feeding beans under drought stress. (**A**) ‘Gongnong535’ fodder soybean; (**B**) ‘Mudanjiang’ fodder soybean. Distinct lowercase letters indicate significant differences (*p* < 0.05) among treatments within the same cultivar and watering condition. Vertical bars denote the standard deviation of the mean (n = 9). Abbreviations and their full forms are provided as follows: NW: normal water supply; DC: drought condition; WT: treated with water; M_50_: treated with 50 μM MT; M_100_: treated with 100 μM MT; M_150_: treated with 150 μM MT.

Drought stress induced or repressed expression of photosynthetic regulation-related genes (*PsaK*, *PsbC*, *Psb27*), antioxidant enzyme genes (*CAT1*, *SOD1*, *POD*), and drought-resistance-related genes (*LEA*, *P5CS*, *DREB*). However, compared with water treatment, MT treatments notably increased the expression levels of these genes to protect ‘Gongnong 535’ and ‘Mudanjiang’ fodder soybean from drought injury, regardless of the concentration of MT used. Peculiarly, the expression of photosynthetic regulation genes *PsaK*, *PsbC* and *Psb27* was most significantly upregulated with the 100 μM melatonin treatment (Figure 9).

### 2.10. Comprehensive Evaluation of Different Melatonin Treatments

To identify the optimal melatonin concentration for alleviating drought stress in fodder soybean, a membership function analysis was conducted on morphological and physiological indicators for both fodder soybean cultivars at the seedling stage. The results indicated that the comprehensive evaluation value for ‘Gongnong 535’ fodder soybean was highest with the 100 μM melatonin treatment in all treatments under normal water and drought conditions, reaching 0.895 and 0.969, respectively (Table S2). For ‘Mudanjiang’ fodder soybean, the membership function values under normal watering and drought conditions for M_50_, M_100_, and M_150_ treatments were 0.544, 0.911, and 0.635 and 0.556, 0.985, and 0.624, respectively. The comprehensive evaluation showed that the effects of different melatonin concentrations on both fodder soybean cultivars ranked as M_100_ > M_150_ > M_50_ (Table S3). Thus, 100 μM is the optimal melatonin concentration to alleviating drought damage for fodder soybean seedlings.

## 3. Discussion

Drought stress can significantly inhibit plant growth, resulting in stunted height and an increased root-to-shoot ratio [28,29]. The experimental studies showed that drought stress inhibited plant height, biomass, and root morphology in fodder soybean. However, the application of various melatonin concentrations partially alleviated negative effects on morphology and physiology. These morphological changes were consistent with the role of exogenous melatonin in lemon verbena (*Lippia citriodora*) and maize seedlings under drought stress [30,31].

A primary reason for the reduction in photosynthesis is non-stomatal factors under drought stress [32], such as destruction of chloroplast structure [33], inhibited activity of photosynthetic enzymes [34], and reduced chlorophyll contents [35]. These reactions ultimately lead to negative effects on the plant’s photosynthetic parameters. Photosynthetic efficiency is closely related to the photosynthetic pigment [36]. Drought inhibits chlorophyll synthesis and accelerates its degradation, leading to decreased chlorophyll content [37,38] and energy conversion efficiency of PS I [39,40]. In this work, drought stress led to decreases in chlorophyll a, chlorophyll b, and total chlorophyll contents, along with reductions in Pn, Tr, Gs and Ci. However, melatonin application increased chlorophyll content, mitigated drought-induced chlorophyll loss and significantly improved photosynthetic capacity in fodder soybean seedlings. This aligns with previous findings that 100 μM exogenous melatonin enhanced total chlorophyll content and improved photosynthetic parameters in maize seedlings, ultimately increasing photosynthetic efficiency [41,42]. We speculated that exogenous melatonin may decrease chloroplast degradation, increase chlorophyll content as well as light absorption efficiency, and improve photosynthetic efficiency, thereby promoting growth and development of fodder soybean under drought stress. Moreover, PS II plays an important role in the light energy conversion and electron transport processes of photosynthesis [43,44], and chlorophyll fluorescence parameters indicate energy conversion efficiency of PS II, commonly used to monitor the degree of photoinhibition in plants under stress conditions [12,45]. In this test, drought stress notably reduced Fv/Fm, ETR, and qP in fodder soybean, indicating damage to the photosynthetic system. However, melatonin application at different concentrations significantly improved fluorescence parameters, effectively alleviating drought-induced damage to the photosynthetic system. This is consistent with findings in fenugreek (*Trigonella foenum-graecum* L.) [46]. Correspondingly, the expression levels of photosynthetic regulation-related genes (*PsaK*, *PsbC*, *Psb27*) were also significantly elevated. Thus, we propose that melatonin enhances drought tolerance in fodder soybean by improving light absorption, electron transport rates, and energy conversion efficiency within the photosystem, while also upregulating related gene expression to elevate photosynthetic efficiency under drought stress.

Osmotic adjustment substances play a vital role in maintaining turgor pressure inside and outside plant cells under drought stress, stabilizing cell and protein structures, ensuring plant stomata open and close normally, and sustaining CO_2_ intake along with the ability of cells to absorb or retain water. These functions help to maintain the normal operation of the photosynthetic system and cellular physiological metabolism, thereby protecting the metabolic processes within the plant [47,48,49,50]. Studies on drought tolerance in wheel wingnut [*Cyclocarya paliurus* (Batal.) Iljinskaja] indicate that an increase in osmotic adjustment substances within the plant enhances osmotic adjustment capacity, thereby improving drought tolerance [51]. However, the osmotic adjustment capacity of plants is limited, particularly under severe drought stress, where cell membranes are prone to severe damage [52]. Exogenous substances can effectively regulate osmotic adjustment substance content [53]. In studies on rice seedlings exposed to drought stress, the application of exogenous melatonin was found to elevate Pro and SS levels, thereby enhancing osmotic adjustment capacity and subsequently improving drought resistance [19]. In this experiment, the application of different concentrations of melatonin under drought stress increased SS, SP, and Pro contents in fodder soybean, while MDA content and REC decreased. It is hypothesized that exogenous melatonin enhanced the osmotic adjustment capacity in fodder soybean seedlings, maintained cell membrane permeability, and slowed stomatal closure, thus facilitating CO_2_ intake. Additionally, adjusting stomatal opening and closing by osmotic adjustment substance content contributed to the improvement of CO_2_ absorption capacity, which may be one of the reasons why exogenous melatonin improves the photosynthetic capacity of fodder soybeans under drought stress.

The production of ROS within plants under drought stress significantly increases, and excessive accumulation leads to oxidative damage to cell membranes. This damage results in lipid peroxidation, thereby decreasing membrane fluidity, increasing permeability, and impairing membrane function [54,55,56,57]. While the ascorbate–glutathione (AsA-GSH) cycle is a non-enzymatic system for ROS scavenging, it plays a crucial role in boosting drought tolerance in plants [58]. In this observation, melatonin application under drought condition remarkably increased AsA and GSH content, which was conducive to enhancing the activity of the AsA-GSH cycle, effectively scavenging excess ROS. In addition, enzymatic antioxidants such as SOD, POD and CAT are also important for scavenging ROS [59]. The SOD catalyzes the dismutation of O_2_^−^ into H_2_O_2_ and O_2_, while POD and CAT help eliminate H_2_O_2_ [60,61]. Under drought conditions, O_2_^−^ and H_2_O_2_ content in fodder soybean leaves significantly increased. After melatonin application, the activities of SOD, POD and CAT were elevated, and ROS content was reduced. Additionally, the expression levels of antioxidant enzyme genes (*CAT1*, *SOD1*, *POD*) were upregulated. This suggests that melatonin mitigated the excessive ROS accumulation caused by drought through enhancing both the non-enzymatic and enzymatic antioxidants and expression level of related genes. Then, melatonin alleviated ROS-induced damage to cell membranes of fodder soybean seedlings under drought stress, which is similar to the results of studies on tomato (*Solanum lycopersicum* L.) drought resistance [62].

## 4. Materials and Methods

### 4.1. Plant Material and Treatments

The experiment was conducted in December 2023 at the Horticultural Experiment Station of Northeast Agricultural University (E 126°14′; N 45°05′). Two fodder soybean cultivars commonly grown in northeast China, ‘Gongnong 535’ and ‘Mudanjiang’, were selected as materials. Seeds of uniform size and plumpness were sown in PVC seedling pots (top diameter 16 cm, bottom diameter 10 cm, height 20 cm) containing 600 g substrate with the 2∶1 volume ratio of soil to vermiculite. Ten seeds were sown per pot, with the pots kept in a greenhouse at 25 °C ± 5 °C, under a light intensity of 1000 ± 50 µmol·m⁻^2^·s⁻^1^, a 16/8 h light/dark photoperiod, and a relative humidity of 75% ± 5%. Deionized water was poured every two days until the fodder soybean seedlings reached the V3 stage, and then, treatments began.

The experiment treatments included the normal water supply group and drought stress group. Each group was treated with water and different concentrations of melatonin (50 μM, 100 μM, 150 μM) on both sides of the leaves of fodder soybean seedlings at 20:00 every day. The treatment standard was that droplets covered both sides of the leaf surfaces without dripping (40 mL per pot). There was a total of 8 experimental treatments, with 5 pots for each treatment. Drought conditions were achieved by artificially regulating the frequency and amount of irrigation. When the relative water content of soil reached 30% using the weighing method, the treatments was stopped, and phenotypic characteristics were observed and photosynthetic parameters were measured. Leaf samples of plants were harvested, promptly frozen in liquid nitrogen, and stored at −80 °C for subsequent analysis of physiological indexes and gene expression levels.

### 4.2. Measurement of Morphological Indexes

The aboveground and belowground parts of each treatment were separated using a sterilized scalpel. The roots of the plant were rinsed with distilled water, and the clean roots were laid flat on the root scanner (LA-S, Wanshen, Zhejiang, China) to measure root morphology, root length, root surface area, and average root diameter [63]. Aboveground and belowground biomass was measured by weighing with analytical balance (FA-C, Techcomp, Shanghai, China) [63]. Each index per treatment was repeated 5 times.

### 4.3. Measurement of Photosynthetic Related Indexes

From 9:00 to 11:00 a.m., the third leaf from the top of each plant was selected to measure net photosynthetic rate (Pn), transpiration rate (Tr), stomatal conductance (Gs), and intercellular CO_2_ concentration (Ci) using a portable photosynthesis system (Li-6400 XT, LiCORInc., Lincoln, NE, USA) [63]. Chlorophyll content was determined using a 95% ethyl alcohol extraction method [64]. Each indicator per treatment was repeated 3 times.

After a 30 min dark adaptation, plants were placed in a chlorophyll fluorescence imaging system (IMAGING-PAM, Heinz Walz GmbH, Nuremberg, BY, Germany) to measure Chlorophyll Fluorescence Parameter, including the photochemical quenching coefficient (qP), non-photochemical quenching coefficient (qN), electron transport rate (ETR), and photosystem II efficiency (Fv/Fm) [63]. Each index per treatment was repeated 3 times.

### 4.4. Measurement of Osmotic Adjustment and Membrane Lipid Peroxidation Indexes

The soluble sugar (SS) content was measured using the anthrone colorimetric method [65]. The soluble protein (SP) content was determined by the coomassie brilliant blue method [64]. The free proline (Pro) content was assessed using the sulfosalicylic acid method [66]. The Malondialdehyde (MDA) content was detected by the thiobarbituric acid (TBA) method [67], and the relative electrolyte conductivity (REC) was quantified using a conductivity meter method [68]. Each indicator per treatment was repeated 3 times.

### 4.5. Measurement of ROS, Antioxidant Enzymes, and Non-Enzymatic Antioxidants

The superoxide anion (O_2_^−^) content was measured using the hydroxylamine oxidation method [65], and the hydrogen peroxide (H₂O₂) content was determined using a commercial assay kit (Grace Biotechnology, Suzhou, China). The reduced ascorbic acid (AsA) content was detected following the method of Murshed (2013) [69]. The reduced glutathione (GSH) was quantified by spectrophotometry [65]. The superoxide dismutase (SOD) activity was measured using the nitroblue tetrazolium (NBT) photoreduction method [65]; the peroxidase (POD) activity was assessed by the guaiacol method [65]. The catalase (CAT) activity was determined by the ultraviolet absorption method [65]. Each index per treatment was repeated 3 times.

### 4.6. Gene Expression Analysis by qRT-PCR

Gene expression analysis was conducted using a Quantagene q225 fluorescence quantitative PCR instrument, following the instructions of the ChamQTM Universal SYBR^®^ qPCR Master Mix kit (Vazyme, Nanjing, China). Relative expression levels of genes were calculated using the 2^−△△ct^ method. All leaf samples were analyzed with three biological replicates and three technical replicates. The primers of the qRT-PCR are listed in Table S4.

### 4.7. Statistical Analysis

Data were analyzed using SPSS 26.0 software (IBM, Armonk, NY, USA) for one-way ANOVA; it was determined through Duncan’s test that differences were considered statistically significant at *p* < 0.05. Graphing was conducted with GraphPad Prism v8 (GraphPad Company, La Jolla, CA, USA). Comprehensive evaluation of each index was analyzed using membership functions in fuzzy mathematics, calculated according to the method described by Zhao (2022) [70].

*R*(*X_i_*) = (*X_i_* − *X_min_*)/(*X_max_* − *X_min_*) (1)

*R*(*X_i_*) = 1 − (*X_i_* − *X_min_*)/(*X_max_* − *X_min_*) (2)

In the formula, *X_i_* represents the measured value of indicator *i*, while *X_max_* and *X_min_* denote the maximum and minimum values of a specific indicator across all treatments, respectively. *R(X_i_)* is the membership function value for indicator *X_i_*, physiological indexes positively and negatively correlated with drought resistance were, respectively, used in (1), (2). Finally, the membership function values of all indicators are summed, and the average value is calculated.

## 5. Conclusions

The findings reveal that melatonin application at different concentrations enhanced phenotype, strengthened the antioxidant defense system, and boosted photosynthetic capacity, effectively alleviating the negative impacts of water scarcity on the growth and development of fodder soybean seedlings. Under drought stress, melatonin treatments alleviate drought-induced damage to cell membrane permeability in fodder soybean through the upregulation of antioxidant enzyme activities, increase in AsA and GSH content, scavenging of excessive H_2_O_2_ and O_2_^−^, regulation of osmotic adjustment substances, and significant reductions in MDA content and REC value. Melatonin application further enhanced photosynthetic capacity during drought conditions by increasing chlorophyll content and improving the light energy conversion efficiency as well as electron capture efficiency in PS I and PS II. Moreover, different melatonin concentrations regulated the expression of antioxidant enzymes and photosynthesis-related genes under drought stress. Significantly, 100 µmol·L⁻^1^ melatonin treatment indicates the most pronounced positive impact on growth and physiological metabolism among all treatments in fodder soybean under the drought condition. These findings offer a theoretical foundation for employing exogenous substances to improve drought tolerance in fodder soybean. What is more notable is that further field trials and advanced molecular techniques are essential to unravel the mechanisms underlying melatonin’s role in alleviating drought stress in fodder soybean.

## Figures and Tables

**Table 1:** Effects of different melatonin (MT) concentrations on growth parameters of fodder soybean under drought condition.

Treatments		Plant Height(cm)		Aboveground Biomass(g)		Belowground Biomass(g)	
		Gongnong 535	Mudanjiang	Gongnong 535	Mudanjiang	Gongnong 535	Mudanjiang
Normal Water	WT	31.93 ± 0.97 a	29.27 ± 0.68 b	0.288 ± 0.003 b	0.242 ± 0.005 c	0.041 ± 0.003 a	0.040 ± 0.003 b
	M_50_	32.17 ± 0.35 a	30.67 ± 0.57 a	0.293 ± 0.002 ab	0.258 ± 0.001 b	0.042 ± 0.002 a	0.042 ± 0.001 b
	M_100_	32.97 ± 1.69 a	31.90 ± 0.70 a	0.300 ± 0.005 a	0.277 ± 0.003 a	0.046 ± 0.003 a	0.046 ± 0.002 a
	M_150_	32.60 ± 2.52 a	30.87 ± 0.65 a	0.299 ± 0.009 a	0.263 ± 0.002 b	0.045 ± 0.004 a	0.043 ± 0.001 ab
Drought Condition	WT	28.47 ± 1.81 b	25.87 ± 0.70 b	0.160 ± 0.006 b	0.124 ± 0.002 c	0.036 ± 0.002 b	0.033 ± 0.002 b
	M_50_	29.23 ± 0.60 ab	27.47 ± 1.02 a	0.170 ± 0.005 ab	0.134 ± 0.003b	0.038 ± 0.001 b	0.037 ± 0.002 a
	M_100_	30.70 ± 0.62 a	28.23 ± 0.81 a	0.174 ± 0.004 a	0.143 ± 0.003a	0.040 ± 0.001 a	0.039 ± 0.002 a
	M_150_	29.43 ± 0.60 ab	27.33 ± 0.55 ab	0.167 ± 0.005 ab	0.136 ± 0.003 b	0.038 ± 0.001 ab	0.037 ± 0.002 a

Each data point is marked with lowercase letters to indicate statistical significance. Distinct lowercase letters indicate significant differences (*p* < 0.05) among treatments within the same cultivar and watering condition. WT: treated with water; M_50_: treated with 50 μM MT; M_100_: treated with 100 μM MT; M_150_: treated with 150 μM MT.

**Table 2:** Effects of different concentrations of melatonin on root indexes of fodder soybean under drought condition.

Treatments		Root Length(cm)		Root Surface Area(cm^2^)		Root Mean Diameter(mm)	
		Gongnong 535	Mudanjiang	Gongnong 535	Mudanjiang	Gongnong 535	Mudanjiang
Normal Water	WT	553.69 ± 3.23 a	545.77 ± 12.91 a	64.29 ± 0.21 d	62.06 ± 0.77 c	0.55 ± 0.03 a	0.45 ± 0.03 a
	M_50_	553.49 ± 10.45 a	557.34 ± 9.44 a	66.74 ± 0.51 c	66.35 ± 0.38 a	0.52 ± 0.03 a	0.46 ± 0.01 a
	M_100_	563.02 ± 13.96 a	560.99 ± 7.41 a	68.74 ± 0.27 a	65.76 ± 0.70 a	0.56 ± 0.02 a	0.47 ± 0.03 a
	M_150_	549.41 ± 9.41 a	554.61 ± 11.99 a	67.70 ± 0.49 b	64.49 ± 0.37 b	0.54 ± 0.02 a	0.46 ± 0.05 a
Drought Condition	WT	272.47 ± 8.00 b	265.70 ± 5.30 b	59.92 ± 0.34 c	58.82 ± 0.54 b	0.50 ± 0.01 a	0.41 ± 0.04 a
	M_50_	346.62 ± 4.21 a	351.55 ± 10.34 a	61.65 ± 0.52 ab	59.73 ± 0.67 b	0.51 ± 0.03 a	0.41 ± 0.06 a
	M_100_	366.39 ± 15.59 a	353.44 ± 12.32 a	62.79 ± 0.61 a	61.24 ± 0.47 a	0.55 ± 0.02 a	0.45 ± 0.03 a
	M_150_	345.99 ± 11.77 a	344.05 ± 11.34 a	60.86 ± 1.11 bc	58.86 ± 0.69 b	0.51 ± 0.03 a	0.45 ± 0.04 a

Each data point is marked with lowercase letters to indicate statistical significance. Distinct lowercase letters indicate significant differences (*p* < 0.05) among treatments within the same cultivar and watering condition. WT: treated with water; M_50_: treated with 50 μM MT; M_100_: treated with 100 μM MT; M_150_: treated with 150 μM MT.

## Data Availability

The original contributions presented in this study are included in the article/Supplementary Material; further inquiries can be directed to the corresponding authors.

## Supplementary Materials

The following supporting information can be downloaded at: https://www.mdpi.com/article/10.3390/plants14030460/s1, Table S1: Effects of different melatonin concentrations on the leaf relative water content of fodder soybean under drought condition. Figure S1: Effects of different melatonin concentrations on the phenotype of fodder soybean under drought stress. Table S2: Comprehensive analysis of effects of different concentrations of exogenous melatonin on ‘Gongnong 535’ fodder soybean under drought condition. Table S3: Comprehensive analysis of effects of different concentrations of exogenous melatonin on ‘Mudanjiang’ fodder soybean under drought condition. Table S4: Primers used for qRT-PCR.

## References

[1] Kaukab R., Sowbiya M. (2021). Drought stress-induced physiological mechanisms, signaling pathways and molecular response of chloroplasts in common vegetable crops. Crit. Rev. Biotechnol..

[2] Sadhukhan A., Prasad S.S., Mitra J., Siddiqui N., Sahoo L., Kobayashi Y., Koyama H. (2022). How do plants remember drought. Planta.

[3] Saleem A., Roldán-Ruiz I., Aper J., Muylle H. (2022). Genetic control of tolerance to drought stress in soybean. BMC Plant Biol..

[4] Xu W., Wuyun T., Chen J., Yu S., Zhang X., Zhang L. (2023). Responses of Trollius chinensis to drought stress and rehydration: From photosynthetic physiology to gene expression. Plant Physiol. Biochem..

[5] Mahmood T., Khalid S., Abdullah M., Ahmed Z., Shah M.K.N., Ghafoor A., Du X. (2019). Insights into Drought Stress Signaling in Plants and the Molecular Genetic Basis of Cotton Drought Tolerance. Cells.

[6] Saleem M.H., Wang X., Parveen A., Perveen S., Mehmood S., Fiza S., Ail S., Hussain S., Adnan M., Lqbal N. (2022). Alleviation of drought stress by root-applied thiourea is related to elevated photosynthetic pigments, osmoprotectants, antioxidant enzymes, and tubers yield and suppressed oxidative stress in potatoes cultivars. PeerJ.

[7] Shaffique S., Hussain S., Kang S.M., Imran M., Injamum-Ul-Hoque M., Khan M.A., Lee L.J. (2023). Phytohormonal modulation of the drought stress in soybean: Outlook, research progress, and cross-talk. Front. Plant Sci..

[8] Yu J., Lu J., Cui X., Guo L., Wang Z., Liu Y., Wang F., Qi M., Liu Y., Li T. (2022). Melatonin mediates reactive oxygen species homeostasis via SlCV to regulate leaf senescence in tomato plants. J. Pineal Res..

[9] Wang L., Tanveer M., Wang H., Arnao M.B. (2024). Melatonin as a key regulator in seed germination under abiotic stress. J. Pineal Res..

[10] Arnao M.B., Hernández-Ruiz J. (2020). Melatonin in flowering, fruit set and fruit ripening. Plant Reprod..

[11] Wang M., Zhang T., Ding F. (2019). Exogenous Melatonin Delays Methyl Jasmonate—Triggered Senescence in Tomato Leaves. Agronomy.

[12] Sharma A., Zheng B. (2019). Melatonin Mediated Regulation of Drought Stress: Physiological and Molecular Aspects. Plants.

[13] Gu Q., Xiao Q., Chen Z., Han Y. (2022). Crosstalk between Melatonin and Reactive Oxygen Species in Plant Abiotic Stress Responses: An Update. Int. J. Mol. Sci..

[14] Li R., Yang R., Zheng W., Wu L., Zhang C., Zhang H. (2022). Melatonin Promotes SGT1-Involved Signals to Ameliorate Drought Stress Adaption in Rice. Int. J. Mol. Sci..

[15] Zhu L., Li A., Sun H., Li P., Guo C., Zhang Y., Zhang K., Bai Z., Dong H. (2023). The effect of exogenous melatonin on root growth and lifespan and seed cotton yield under drought stress. Ind. Crops Prod..

[16] Hu W., Zhang J., Yan K., Zhou Z., Zhao W., Zhang X., Pu Y., Yu R. (2021). Beneficial effects of abscisic acid and melatonin in overcoming drought stress in cotton (*Gossypium hirsutum* L.). Physiol. Plant..

[17] Dai L., Li J., Harmens H., Zheng X., Zhang C. (2020). Melatonin enhances drought resistance by regulating leaf stomatal behavior, root growth and catalase activity in two contrasting rapeseed (*Brassica napus* L.) genotypes. Plant Physiol. Biochem..

[18] Kuppusamy A., Alagarswamy S., Karuppusami K.M., Maduraimuthu D., Natesan S., Ramalingam K., Muniyappan U., Subramanian M., Kanagarajan S. (2023). Melatonin Enhances the Photosynthesis and Antioxidant Enzyme Activities of Mung Bean under Drought and High-Temperature Stress Conditions. Plants.

[19] Luo C., Min W., Akhtar M., Lu X., Bai X., Zhang Y., Tian L., Li P. (2022). Melatonin Enhances Drought Tolerance in Rice Seedlings by Modulating Antioxidant Systems, Osmoregulation, and Corresponding Gene Expression. Int. J. Mol. Sci..

[20] El-Yazied A.A., Ibrahim M.F.M., Ibrahim M.A.R., Nasef I.N., Al-Qahtani S.M., Al-Harbi N.A., Alzuaibr F.M., Alaklabi A., Dessoky E.S., Alabdallah N.M. (2022). Melatonin Mitigates Drought Induced Oxidative Stress in Potato Plants through Modulation of Osmolytes, Sugar Metabolism, ABA Homeostasis and Antioxidant Enzymes. Plants.

[21] Su X., Fan X., Shao R., Guo J., Wang Y., Yang J., Yang Q., Guo L. (2019). Physiological and iTRAQ- based proteomic analyses reveal that melatonin alleviates oxidative damage in maize leaves exposed to drought stress. Plant Physiol. Biochem..

[22] Imran M., Latif Khan A., Shahzad R., Aaqil Khan M., Bilal S., Khan A., Kang S.M., Lee I.J. (2021). Exogenous melatonin induces drought stress tolerance by promoting plant growth and antioxidant defense system of soybean plants. AoB Plants.

[23] Cui G., Zhao X., Liu S., Sun F., Zhang C., Xi Y. (2017). Beneficial effects of melatonin in overcoming drought stress in wheat seedlings. Plant Physiol. Biochem..

[24] Ahmad S., Muhammad I., Wang G., Zeeshan M., Yang L., Ali I., Zhou X. (2021). Ameliorative effect of melatonin improves drought tolerance by regulating growth, photosynthetic traits and leaf ultrastructure of maize seedlings. BMC Plant Biol..

[25] Yang M., Wang F., Xu W., Li X., Yin H., Tuluhong M., Qiu R., Li B., Cui G. (2024). Effects of the fermentation quality and microbial community of waxy maize mixed with fodder soybean silage. Front. Microbiol..

[26] Salama H.S.A., Abdel-Moneim M.H. (2021). Maximizing Land Use Efficiency and Productivity of Soybean and Fodder Maize Intercrops through Manipulating Sowing Schedule and Maize Harvest Regime. Agronomy.

[27] Jia J., Wang H., Cai Z., Wei R., Huang J., Xia Q., Xiao X., Ma Q., Nian H., Cheng Y. (2022). Identification and validation of stable and novel quantitative trait loci for pod shattering in soybean [*Glycine max* (L.) Merr.]. J. Integr. Agric..

[28] He X., Xu L., Pan C., Gong C., Wang Y., Liu X., Yu Y. (2020). Drought resistance of Camellia oleifera under drought stress: Changes in physiology and growth characteristics. PLoS ONE.

[29] Yu X., Liu Y., Cao P., Zeng X., Xu B., Luo F., Yang X., Wang X., Wang X., Xiao X. (2023). Morphological Structure and Physiological and Biochemical Responses to Drought Stress of *Iris japonica*. Plants.

[30] Hosseini M.S., Samsampour D., Zahedi S.M., Zamanian K., Rahman M.M., Mostofa M.G., Tran L.P. (2021). Melatonin alleviates drought impact on growth and essential oil yield of lemon verbena by enhancing antioxidant responses, mineral balance, and abscisic acid content. Physiol. Plant.

[31] Ahmad S., Wang G., Muhammad I., Chi Y., Zeeshan M., Nasar J., Zhou B. (2022). Interactive Effects of Melatonin and Nitrogen Improve Drought Tolerance of Maize Seedlings by Regulating Growth and Physiochemical Attributes. Antioxid. (Basel).

[32] Zhang W., Wang L., Zhang L., Kong X., Zhang J., Wang X., Pei Y., Jin Z. (2022). H_2_S-mediated balance regulation of stomatal and non- stomatal factors responding to drought stress in Chinese cabbage. Hortic. Res..

[33] Yang K., Sun H., Liu M., Zhu L., Zhang K., Zhang Y., Li A., Zhang H., Zhu J., Liu X. (2023). Morphological and Physiological Mechanisms of Melatonin on Delaying Drought-Induced Leaf Senescence in Cotton. Int. J. Mol. Sci..

[34] Ramachandra Reddy A., Chaitanya K.V., Vivekanandan M. (2004). Drought-induced responses of photosynthesis and antioxidant metabolism in higher plants. J. Plant Physiol..

[35] Ghobadi M., Taherabadi S., Ghobadi M.E., Mohammadi G.R., Jalali-Honarmand S. (2013). Antioxidant capacity, photosynthetic characteristics and water relations of sunflower (*Helianthus annuus* L.) cultivars in response to drought stress. Ind. Crops Prod..

[36] Wu X., Rayyan K., Gao H., Liu H., Zhang J., Ma X. (2021). Low Light Alters the Photosynthesis Process in Cigar Tobacco via Modulation of the Chlorophyll Content, Chlorophyll Fluorescence, and Gene Expression. Agriculture.

[37] Xu Q., Ma X., Lv T., Bai M., Wang Z., Niu J. (2020). Effects of Water Stress on Fluorescence Parameters and Photosynthetic Characteristics of Drip Irrigation in Rice. Water.

[38] Li C., Tan D., Liang D., Chang C., Jia D., Ma F. (2015). Melatonin mediates the regulation of ABA metabolism, free-radical scavenging, and stomatal behavior in two Malus species under drought stress. J. Exp. Bot..

[39] Shinya W., Daisuke T., Chikahiro M., Amane M., Yuji S. (2019). Responses of the Photosynthetic Electron Transport Reactions Stimulate the Oxidation of the Reaction Center Chlorophyll of Photosystem I, P700, under Drought and High Temperatures in Rice. Int. J. Mol. Sci..

[40] Supriya L., Durgeshwar P., Muthamilarasan M., Padmaja G. (2022). Melatonin Mediated Differential Regulation of Drought Tolerance in Sensitive and Tolerant Varieties of Upland Cotton (*Gossypium hirsutum* L.). Front. Plant Sci..

[41] Ahmad S., Wang G., Muhammad I., Farooq S., Kamran M., Ahmad I., Zeeshan M., Javed T., Ullah S., Huang J. (2022). Application of melatonin-mediated modulation of drought tolerance by regulating photosynthetic efficiency, chloroplast ultrastructure, and endogenous hormones in maize. Chem. Biol. Technol. Agric..

[42] Ye J., Wang S., Deng X., Yin L., Xiong B., Wang X. (2016). Melatonin increased maize (*Zea mays* L.) seedling drought tolerance by alleviating drought- induced photosynthetic inhibition and oxidative damage. Acta Physiol. Plant..

[43] Talaat N.B. (2023). Drought Stress Alleviator Melatonin Reconfigures Water- Stressed Barley (*Hordeum vulgare* L.) Plants’ Photosynthetic Efficiency, Antioxidant Capacity, and Endogenous Phytohormone Profile. Int. J. Mol. Sci..

[44] Moustaka J., Sperdouli I., İşgören S., Sas B., Moustakas M. (2024). Deciphering the Mechanism of Melatonin-Induced Enhancement of Photosystem II Function in Moderate Drought-Stressed Oregano Plants. Plants.

[45] Liu T., Sheng M., Wang Y., Chen H., Li Z., Tang M. (2015). Impact of arbuscular mycorrhizal fungi on the growth, water status, and photosynthesis of hybrid poplar under drought stress and recovery. Photosynthetica.

[46] Maleki M., Shojaeiyan A., Mokhtassi-Bidgoli A., Tamadoni-Saray M. (2024). Melatonin Enhances Drought Tolerance and Recovery Capability in Two Contrasting Fenugreek (*Trigonella foenum-graecum* L.) Landraces Through Improved Photosynthetic Apparatus Protection and Carboxylation Efficiency. J. Plant Growth Regul..

[47] Muhammad I., Yang L., Ahmad S., Farooq S., Khan A., Muhammad N., Ullah S., Adnan M., Ali S., Liang Q. (2023). Melatonin-priming enhances maize seedling drought tolerance by regulating the antioxidant defense system. Plant Physiol..

[48] He M., Mei S., Zhai Y., Geng G., Yu L., Wang Y. (2022). Effects of Melatonin on the Growth of Sugar Beet (*Beta vulgaris* L.) Seedlings Under Drought Stress. J. Plant Growth Regul..

[49] Wang B., Li X., Wang G. (2023). Responses of the desert green algae, Chlorella sp. to drought stress. J. Phycol..

[50] Ozturk M., Unal B.T., García-Caparrós P., Khursheed A., Gul A., Hasanuzzaman M. (2021). Osmoregulation and its actions during the drought stress in plants. Physiol. Plant..

[51] Li C., Wan Y., Shang X., Fang S. (2023). Integration of transcriptomic and metabolomic analysis unveils the response mechanism of sugar metabolism in *Cyclocarya paliurus* seedlings subjected to PEG-induced drought stress. Plant Physiol. Biochem..

[52] Ye Q., Li L., Jacobs D.F., Li M., Peng L., Song H., Jia S. (2015). Physiological response to drought stress in *Camptotheca acuminata* seedlings from two provenances. Front. Plant Sci..

[53] Zali A.G., Ehsanzadeh P. (2018). Exogenous proline improves osmoregulation, physiological functions, essential oil, and seed yield of fennel. Ind. Crops Prod..

[54] Xiong J., Zhang W., Zheng D., Xiong H., Feng X., Zhang X., Wang Q., Wu F., Xu J., Lu Y. (2022). *ZmLBD5* Increases Drought Sensitivity by Suppressing ROS Accumulation in Arabidopsis. Plant.

[55] Lv X., Li Y., Chen R., Chen R., Rui M., Wang Y. (2023). Stomatal Responses of Two Drought-Tolerant Barley Varieties with Different ROS Regulation Strategies under Drought Conditions. Antioxid..

[56] Fang Y., Xiong L. (2015). General mechanisms of drought response and their application in drought resistance improvement in plants. Cell. Mol. Life Sci..

[57] Abbas K., Li J., Gong B., Lu Y., Wu X., Lv G., Gao H. (2023). Drought Stress Tolerance in Vegetables: The Functional Role of Structural Features, Key Gene Pathways, and Exogenous Hormones. Int. J. Mol. Sci..

[58] Jiang Z., Zhu H., Zhu H., Tao Y., Liu C., Liu J., Yang F., Li M. (2022). Exogenous ABA Enhances the Antioxidant Defense System of Maize by Regulating the AsA-GSH Cycle under Drought Stress. Sustainability.

[59] Zhang P., Hu Y., Zhou R., Zhang X., Hu H., Lang D. (2022). The antioxidant system response to drought-stressed *Diospyros lotus* treated with exogenous melatonin. PeerJ.

[60] Muhammad I., Li Y., Ahmad S., Mosaad I.S.M., Al-Ghamdi A.A., Abbasi A.M., Zhou X. (2022). Melatonin Application Alleviates Stress-Induced Photosynthetic Inhibition and Oxidative Damage by Regulating Antioxidant Defense System of Maize: A Meta-Analysis. Antioxidants.

[61] Guo Y., Li D., Liu L., Sun H., Zhu L., Zhang K., Zhao H., Zhang Y., Li A., Bai Z. (2022). Seed Priming with Melatonin Promotes Seed Germination and Seedling Growth of *Triticale hexaploide* L. Under PEG-6000 Induced Drought Stress. Front. Plant Sci..

[62] Altaf M.A., Shahid R., Ren M., Naz S., Altaf M.M., Khan L.U., Tiwari R.K., Lal M.K., Shahid M.A., Kumar R. (2022). Melatonin Improves Drought Stress Tolerance of Tomato by Modulating Plant Growth, Root Architecture, Photosynthesis, and Antioxidant Defense System. Antioxidants.

[63] Xie F., Liu Y., Zhao Q., Liu X., Wang C., Wang Q., Wei Q., Zhao X., Jiang J., Liu R. (2024). Exogenous Application of Melatonin and Strigolactone by Regulating Morphophysiological Responses and Gene Expression to Improve Drought Resistance in Fodder Soybean Seedlings. Agronomy.

[64] Zhang Y., Cheng W., Di H., Yang S., Tian Y., Tong Y., Huang H., Escalona V.H., Tang Y., Li H. (2024). Variation in Nutritional Components and Antioxidant Capacity of Different Cultivars and Organs of *Basella alba*. Plants.

[65] Gao J. (2006). Experimental guidance for plant physiology.

[66] Abrahám E., Hourton-Cabassa C., Erdei L., Szabados L. (2010). Methods for Determination of Proline in Plants. Plant Stress Toler..

[67] Cowan I.R., Farquhar G.D. (1977). Stomatal Function in Relation to Leaf Metabolism and Environment: Stomatal Function in the Regulation of Gas Exchange. Symp. Soc. Exp. Biol..

[68] Zhao X., Tian Z., Cheng L., Jiang J., Liu Y., Liu L., You C., Liu X., Xie F., Qin L. (2023). Comparative Study on the Morpho-Physiological Responses of White Clover Cultivars with Different Leaf Types to Water Deficiency. Agronomy.

[69] Murshed R., Lopez-Lauri F., Sallanon H. (2013). Effect of water stress on antioxidant systems and oxidative parameters in fruits of tomato (*Solanum lycopersicon* L., cv. Micro-tom). Physiol. Mol. Biol. Plants..

[70] Zhao H., Wu R., Liu Z., Liu Z., Liu G. (2022). Evaluation of salt tolerance during the seed germination stage of five varieties of *Vicia sativa*. Legume Res..

