# Supplementary material for: Exogenous Melatonin Improves Drought Tolerance by Regulating the Antioxidant Defense System and Photosynthetic Efficiency in Fodder Soybean Seedings
Source: Plants (Basel). 2025 Feb 5;14(3):460. doi: 10.3390/plants14030460 (PMC11819762; doi:10.3390/plants14030460)
Supplement: Supplementary file 1 [file plants-14-00460-s001.zip › plants-3421974-supplementary.pdf]

Supplemental Materials

Table S1 Effects of different melatonin concentrations on the leaf relative water content of fodder soybean under drought condition (%)

Treatments		Gongnong 535	Mudanjiang
Normal Water	WT	74.80±0.009b	74.05±0.003b
	M_{50}	74.91±0.009b	74.84±0.001ab
	M_{100}	75.43±0.003a	75.58±0.009a
	M_{150}	78.73±0.007a	74.12±0.008b
Drought Condition	WT	33.89±0.005b	32.72±0.010c
	M_{50}	34.33±0.007b	32.07±0.008c
	M_{100}	37.54±0.006a	37.23±0.009a
	M_{150}	36.83±0.003b	34.77±0.010b

Each data point is marked with lowercase letters to indicate statistical significance. Distinct lowercase letters indicate significant differences ($p < 0.05$) among treatments within the same cultivar and watering condition. WT: treated with water; M50: treated with 50 μM MT; M100: treated with 100 μM MT; M150: treated with 150 μM MT

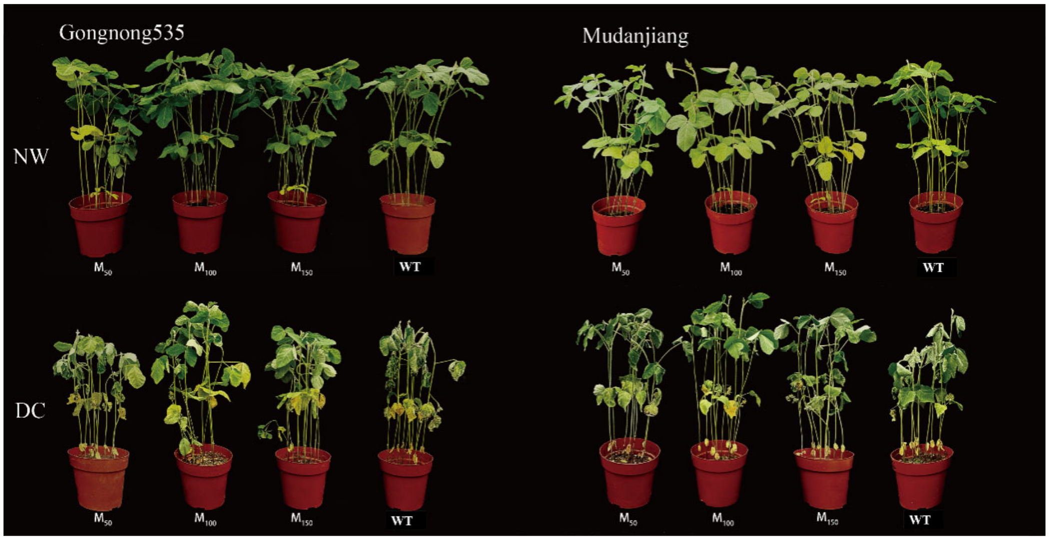

Figure S1 Effects of different melatonin concentrations on the phenotype of fodder soybean under drought stress. (A) 'Gongnong 535' fodder soybean fodder soybean; (B) 'Mudanjiang' fodder soybean. Abbreviations and corresponding full names are as follows: NW: normal water supply; DC: drought condition; WT: treated with water; M_{50}: treated with 50 μM MT; M_{100}: treated with 100 μM MT; M_{150}: treated with 150 μM MT.

Table S2 Comprehensive analysis of effects of different concentrations of exogenous melatonin on 'Gongnong 535' fodder soybean under drought condition

Index	NW				DC			
	WT	M_{50}	M_{100}	M_{150}	WT	M_{50}	M_{100}	M_{150}
Plant Height	0.000	0.532	1.000	0.608	0.000	1.000	1.000	0.917
Aboveground Biomass	0.000	0.217	1.000	0.597	0.000	0.491	1.000	0.596

Belowground Biomass	0.000	0.412	1.000	0.529	0.000	0.550	1.000	0.650
Root Length	0.314	0.300	1.000	0.000	0.000	0.271	0.343	1.000
Root Surface Area	0.000	0.551	1.000	0.766	0.000	0.603	1.000	0.328
Root Mean Diameter	0.750	0.000	1.000	0.500	0.000	0.200	1.000	0.200
Chlorophyll a content	0.000	0.576	1.000	0.860	0.000	0.672	1.000	0.056
Chlorophyll b content	0.000	0.881	1.000	0.053	0.000	0.295	0.911	1.000
Total chlorophyll content	0.000	0.784	1.000	0.308	0.000	0.566	1.000	0.374
Net photosynthetic rate	0.000	1.000	0.543	0.053	0.000	0.367	1.000	0.673
Stomatal conductance	0.000	0.525	1.000	0.853	0.000	0.311	1.000	0.421
Intercellular CO_2 concentration	0.000	0.497	1.000	0.529	0.000	0.595	1.000	0.615
Transpiration rate	0.000	0.306	1.000	0.446	0.000	0.586	1.000	0.725
ETR	0.000	0.917	0.785	1.000	0.000	1.000	1.000	0.140
qP	0.000	0.536	1.000	0.373	0.938	0.730	1.000	0.000
qN	0.000	0.464	1.000	0.420	0.699	0.328	1.000	0.000
Fv/Fm	0.697	0.000	0.562	1.000	0.000	0.651	1.000	0.718
Soluble sugar content	0.000	0.453	1.000	0.433	0.000	1.000	0.874	0.410
Free Proline content	0.482	0.808	1.000	0.000	0.000	0.682	1.000	0.916
Soluble protein content	0.000	0.471	1.000	0.636	0.000	0.542	1.000	0.873
MDA content	0.886	1.000	0.000	0.043	0.000	0.915	1.000	0.460
Relative electrical conductivity	0.000	0.683	1.000	0.575	0.000	0.458	1.000	0.638
H_2O_2 content	0.000	0.689	1.000	0.803	0.000	0.625	1.000	0.655
$O_2^{\cdot-}$ content	0.000	0.273	0.420	1.000	0.000	0.896	0.968	1.000
Ascorbic acid content	0.000	1.000	0.904	0.681	0.000	0.676	1.000	0.926
Glutathione content	0.000	0.707	1.000	0.439	0.000	0.939	1.000	0.907
SOD activity	0.000	0.910	0.754	1.000	0.000	0.874	1.000	0.943
POD activity	0.000	0.888	1.000	0.817	0.000	0.762	1.000	0.814
CAT activity	0.000	0.309	1.000	0.441	0.000	0.817	1.000	0.882
Mean membership index	0.108	0.575	0.895	0.544	0.056	0.635	0.969	0.615
Rank	4	2	1	3	4	2	1	3

Abbreviations and corresponding full names are as follows: NW: normal water supply; DC: drought condition; WT: treated with water; M_{50}: treated with 50 μM MT; M_{100}: treated with 100 μM MT; M_{150}: treated with 150 μM MT.

Table S3 Comprehensive analysis of effects of different concentrations of exogenous melatonin on 'Mudanjiang' fodder soybean under drought condition

Index	NW				DC			
	WT	M_{50}	M_{100}	M_{150}	WT	M_{50}	M_{100}	M_{150}
Plant Height	0.000	0.226	1.000	0.645	0.000	0.343	1.000	0.433
Aboveground Biomass	0.000	0.389	1.000	0.889	0.000	0.682	1.000	0.500
Belowground Biomass	0.000	0.154	1.000	0.769	0.000	0.417	1.000	0.500
Root Length	0.000	0.760	1.000	0.581	0.000	0.978	1.000	0.893
Root Surface Area	0.000	1.000	0.862	0.566	0.000	0.376	1.000	0.017
Root Mean Diameter	0.000	0.500	1.000	0.500	0.000	0.000	1.000	1.000
Chlorophyll a content	0.000	0.909	1.000	0.783	0.000	1.000	0.911	0.250

Chlorophyll b content	0.342	0.000	0.694	1.000	0.095	0.000	1.000	0.842
Total chlorophyll content	0.000	0.659	1.000	0.928	0.000	0.272	1.000	0.653
Net photosynthetic rate	0.000	1.000	0.838	0.344	0.000	0.638	0.943	1.000
Stomatal conductance	0.000	0.162	1.000	0.607	0.000	0.322	1.000	0.542
Intercellular CO_2 concentration	0.000	0.718	1.000	0.349	0.000	0.711	1.000	0.394
Transpiration rate	0.000	0.382	1.000	0.796	0.000	0.493	1.000	0.717
ETR	0.000	0.264	1.000	0.736	0.000	0.466	1.000	0.634
qP	0.471	0.000	1.000	0.595	0.000	0.181	1.000	0.930
qN	0.094	1.000	0.013	0.000	0.000	0.144	1.000	0.269
Fv/Fm	0.289	0.647	1.000	0.000	0.699	0.142	1.000	0.000
Soluble sugar content	0.000	0.924	1.000	0.941	0.000	1.000	0.805	0.735
Free Proline content	0.644	1.000	0.000	0.021	0.000	0.413	1.000	0.590
Soluble protein content	0.000	0.447	1.000	0.803	0.000	0.563	1.000	0.871
MDA content	0.000	0.971	1.000	0.015	0.000	0.769	1.000	0.773
Relative electrical conductivity	0.000	0.700	1.000	0.800	0.000	0.817	1.000	0.649
H_2O_2 content	0.000	0.510	1.000	0.686	0.000	0.624	1.000	0.599
$O_2^{\cdot-}$ content	0.314	0.000	1.000	0.994	0.000	1.000	0.894	0.835
Ascorbic acid content	0.000	0.469	1.000	0.988	0.000	0.612	1.000	0.831
Glutathione content	0.000	0.476	1.000	0.789	0.000	0.625	1.000	0.827
SOD activity	0.000	0.528	1.000	0.803	0.679	0.933	1.000	0.000
POD activity	0.000	0.352	1.000	0.613	0.000	0.786	1.000	0.922
CAT activity	0.000	0.639	1.000	0.887	0.000	0.822	1.000	0.876
Mean membership index	0.074	0.544	0.911	0.635	0.051	0.556	0.985	0.624
Rank	4	3	1	2	4	3	1	2

Abbreviations and corresponding full names are as follows: NW: normal water supply; DC: drought condition; WT: treated with water; M_{50}: treated with 50 μM MT; M_{100}: treated with 100 μM MT; M_{150}: treated with 150 μM MT.

Table S4 Primers used for qRT-PCR

Gene Name	Upper primer	Lower primer
P5CS	TTGTGATGGAGCACGCTTTG	AACCAAGACCAGATGAGAAAATGAA
LEA	CGAAGCTAAGTTAGTGCGGT	TGGGCGTGGGTTATGTTTC
DREB	GAACAAGGAAGTGAAGGTAGCAA	TTATCTCAGCGGTTGGTGGTT
CAT1	CAGGCATATGGATGGCTTCG	GAGACTTTTCGCCAGAGGTG
SOD1	CCTCTCACTGGACCAAACTCC	AGCTCATGACCACCTTTTCCA
POD	TTGGTTCCGAGCTCTCTCAAG	TCCGAACAAGGGCACTTCAA
PsaK	TCCAACAACCTATGAGGCCC	TCCAACAACCTATGAGGCCC
PsbC	TAACTGCCCTGACTATGGCG	CTGCTTGGACGAAGCGAAAG
Psb27	TTGTCCATCATCACCACCACAT	TCACGATTGTCGTCCTCACTTT
